# Effectiveness of self-managed home and community exercise interventions in improving physical activity, body adiposity and related health indices in adults living with HIV: a protocol for a systematic review

**DOI:** 10.1186/s13643-022-01908-5

**Published:** 2022-03-03

**Authors:** Jeannine Anyingu Aminde, Neil Harris, Caroline Thng, Ben Desbrow

**Affiliations:** 1 School of Health Sciences and Social Work (SHS), Griffith University, Gold Coast, Queensland 4222 Australia; 2 School of Medicine and Dentistry, Griffith University, Gold Coast, Queensland Australia; 3 Gold Coast University Hospital, Gold Coast, Queensland Australia

**Keywords:** Adiposity, Physical activity, Home exercise, Fat distribution, HIV

## Abstract

**Background and objectives:**

Disorders of adipose tissue distribution in people living with the human immunodeficiency virus (HIV) have been associated with significant metabolic derangements that increase their risk of cardiometabolic and other chronic diseases. Systematic reviews focusing on supervised laboratory-based exercise interventions demonstrate that these interventions improve adipose tissue distribution and related health outcomes in people living with HIV (PLWH). However, there is a need to examine the effectiveness of more pragmatic home or community exercise programmes. The aim of this review will be to synthesize existing evidence on the effectiveness of self-managed home or community exercise interventions to improve physical activity levels, adipose tissue distribution and associated health indices in PLWH.

**Methods:**

This review will encompass interventional studies that evaluate the effect of prescribed exercise programmes performed in the home or community with minimal supervision, by adults living with HIV. The following will be searched from inception: PubMed, Embase, Scopus, Cumulative Index to Nursing and Allied Health Literature, Physiotherapy Evidence Database, SPORTDiscus, Cochrane Central Register of Controlled Trials, Cochrane Database of Systematic Reviews and Clinicaltrials.gov. Screening of studies and data extraction will be conducted by two independent reviewers. The risk of bias in included studies will be assessed using version 2 of the Cochrane risk-of-bias tool for randomized trials (RoB 2) and the Risk of Bias In Non-Randomized Studies-of Interventions (ROBINS-I) tool for non-randomized concurrently controlled and single-arm interventional studies. A random effects meta-analysis will be used to pool effect estimates for outcomes of interest (measures of physical activity and body adiposity). However, if pooling is deemed inappropriate due to substantial differences between studies, a narrative synthesis will be performed. This protocol is written according to the Preferred Reporting Items for Systematic reviews and Meta-analysis Protocols 2015 statement (see Additional file 1).

**Discussion:**

This review shall provide evidence to support or disapprove the prescription of self-managed exercise interventions in a particularly vulnerable population. We will equally explore the potential impact of technology in improving physical activity outcomes. Our findings could help guide clinicians involved in the care of PLWH in prescribing exercise and inform the design of future trials and research.

**Systematic review registration:**

PROSPERO CRD42021223357.

**Supplementary Information:**

The online version contains supplementary material available at 10.1186/s13643-022-01908-5.

## Introduction

### Rationale

Disorders of adipose tissue distribution in people living with HIV (PLWH) have garnered fresh interest in recent times. In the pre-antiretroviral therapy (pre-ART) era, it was characterized by HIV wasting [1], which was replaced by lipoatrophy in the early combination ART years associated with the first generation of thymidine analogue drugs such as zidovudine and stavudine [1, 2], as well as early protein inhibitors [1, 3]. However, interest waned as newer generations of antiretrovirals with improved profiles were introduced [1, 4]. In the last decade, the addition of a new class of antiretrovirals which has both improved survival [5] (hence increased the burden of age-related metabolic disorders in PLWH) and appears to induce weight gain [6, 7] has brought adipose tissue redistribution back to the forefront of HIV research.

Inflammatory and immune pathways (which persist even in the virally suppressed) accentuated by antiretroviral-induced cytokine dysregulation are believed to provoke the expansion of metabolically active adipose deposits and adipose redistribution [8–10]. The consequences of which include overweight/obesity [7], insulin resistance [11], dyslipidaemias [11], atherogenesis [12, 13] and oncogenesis [14]. Importantly, these HIV-related factors are aggravated by various lifestyle factors which are significantly more prevalent in the HIV population such as alcohol and substance abuse disorders, smoking, hepatitis B and hepatitis C [15, 16], hence the increased risk of strokes, myocardial infarctions, diabetes mellitus and certain cancers in PLWH [15, 17, 18]. Therefore, strategies that address adiposity disorders and their associated cardiometabolic risk could help mitigate the burden of varied comorbidities in PLWH as they age.

Physical activity reduces inflammation in chronic HIV and is considered a cornerstone in the management of HIV-associated cardiometabolic derangements [8, 19]. Various centre-based/laboratory-based clinical trials have demonstrated that exercise programmes incorporating aerobic and strength training are safe [20–22] and efficacious [20, 23, 24] in correcting adiposity derangements and dyslipidaemias in PLWH. In addition to these benefits, aerobic exercise for PLWH equally improves cardiorespiratory function and quality of life [20, 24], while resistance exercise improves muscle strength [21, 25], immune function [26] and muscle mass [21].

However, the transferability of these benefits to everyday settings remains a challenge. Studies on exercise interventions in PLWH record dropout rates as high as 29% [27]. While professional supervision appears to reduce dropout [27], exercising with an exercise physiologist, sports therapist, physiotherapist or other professionals in an exercise facility is resource intensive. This raises issues of accessibility and long-term sustainability in a population that already faces more frequent hospital visits and higher health care costs [15]. There is therefore the need to assess other exercise options.

Some reviews demonstrate the benefits of home exercise programmes as a viable alternative to centre-based exercise in other chronic conditions [28, 29]. Roos and colleagues found that a home exercise walking programme without supervision reduced waist-to-hip ratio and ischaemic heart disease risk in PLWH [30]. On the other hand, Bonato and colleagues reported significant reductions in fat mass of home exercises only if supported with a mobile application [31]. It is possible that self-managed exercise at home or in the local community with or without (mobile/computer) technology support could prove useful in managing PLWH. To date, no study has synthesized the evidence on home and community-based exercise interventions in PLWH.

Therefore, the aim of this review is to systematically synthesize all available evidence on the effectiveness of home and community exercise interventions with minimal to no supervision on physical activity levels, indices of adiposity and related health status indices in people living with HIV. We equally wish to elucidate the impact of technology support on the efficacy of such programmes.

### Goal

To assess the effects of home and community exercise for physical activity levels, indices of adiposity and related health status indices in adults people living with HIV.

### Objectives

i. (Primary) To determine the efficacy of home and community exercise programmes in improving physical activity levels in adults living with HIV
ii. (Primary) To determine the efficacy of home and community exercise programmes in improving indices of adiposity in adults living with HIV
iii. (Secondary) To determine the efficacy of home and community exercise programmes in improving health status (physical performance, cardiometabolic outcomes and overall wellbeing) in adults living with HIV
iv. (Secondary) To ascertain the impact of technology-assisted remote support (that is through phone, computer or other smart devices) on physical activity levels following home and community exercise programmes in adults living with HIV

## Methods

### Eligibility criteria

#### Population of interest

HIV-positive male or female adults, 18 years and older, at all stages of infection (CD4, viral loads), with or without comorbidities in any part of the world.

#### Intervention

Prescribed exercise performed at home or in the community of at least 4-week duration, with minimal or no supervision. The programme must include either an aerobic component or a resistance component.

We will consider “prescribed exercise” to be a structured physical activity plan made available to participants with specified type, intensity and volume, accompanied by some form of monitoring (such as an exercise diary).

“Community” will refer to local public and outdoor spaces that are freely accessible, such as parks and public recreational facilities (see Table 1).

**Table 1:** Criteria and definition of home/community exercise

Criterion	Home/community minimally supervised	Centre-based supervised
**Supervision**	- Mostly self-managed - Instruction offered during less than half of exercise time	- Mostly supervised - Instruction offered during half or more of exercise time
**Location**	Home environment or local neighbourhood	Within a specialized exercise facility
**Equipment**	Present or absent	Specialized equipment designed for exercise
**Accessibility/cost**	Accessible to the public at little or no cost	Not routinely open to the public
**Examples**	Within home, public outdoor spaces such as parks, community centres, public recreational facilities	Hospital clinics, exercise laboratories, private practice, physiotherapy clinics

We will exclude studies of interventions in a detention or prison facility.

“Minimal supervision” will be regarded as professional input (in-person or virtual) in the form of instruction during exercise sessions for less than half the total duration of exercise time. Remote observation through physical activity monitors or exercise logbooks will not be considered supervision. Professional support restricted to safety or compliance checks, reminders, counselling or motivation will also not be considered supervision.

Aerobic exercise will be defined as any physical activity performed with the aim of strengthening the heart and lungs that results in increased breath and heart rates. This will include (but not limited to) jogging, running, walking, swimming, stair climbing, stepping, rowing, jump rope, dancing and cycling.

Resistance exercise will be defined as any physical activity performed with the aim of strengthening muscle, utilizing muscle contractions against resistance aided by weights (free weights, weight stations amongst others) or unaided (resistance provided by body’s own weight).

#### Comparators

We will compare home/community exercise outcomes to “before exercise” in single-group design or to control arms with no exposure/intervention, standard care, other types of exercise programmes such as centre-based exercise (see Table 1) or other therapeutic modalities.

#### Outcomes

##### Primary

Our primary outcomes shall include the following measures (the classification of adiposity measures was adapted from previous work [32]):

i. *Total body adiposity*

- Total fat mass
- Percent body fat
- Body mass index ((BMI = body weight in kilogrammes/(height in metres)^2^)
- Fat mass index ((FMI = total fat mass in kilogrammes/(height in metres)^2^) [33]
- Body adiposity index (BAI = ((hip circumference in centimetres)/((height in metres)^1.5^)–18)) [34]

ii. *Body adipose tissue distribution*

- Waist-to-hip ratio (WHR)
- Waist-to-height ratio
- Waist-to-thigh ratio
- Supine sagittal abdominal diameter (abdominal anteroposterior diameter in a supine person measured directly or by imaging) [32]
- Waist circumference

iii. *Regional adiposity*

- Skinfold thickness measurements
- Visceral adipose tissue (VAT) volumes/mass
- Regional fat percentages
- Subcutaneous fat volumes/mass

iv. *Physical activity levels (self-report or device measured)*

In this study, weight will not be considered a primary outcome measure of adiposity because of its well-recognized inability to account for body build and its susceptibility to changes in lean mass [32].

##### Secondary

In addition to the above primary outcomes, the following will be evaluated:

i. *Physical performance*

- Muscle/lean mass and strength
- Disability
- Self-efficacy

ii. *Cardiometabolic outcomes*

- Metabolic parameters (fasting glucose, lipids)
- Cardiorespiratory fitness

iii. *Overall health status and wellbeing*

- Changes in weight
- Virologic outcomes (viral load, CD4+ T cells)
- Quality of life measures
- Psychological health measures
- Adverse events related to exercise

iv. *Adherence to exercise programme*

#### Study characteristics

Randomized controlled trials, non-randomized controlled trials and uncontrolled single-arm (pre-post) interventional studies will be included. This choice was made after pre-review scoping of the literature revealed a scarcity of comparative studies and more so randomized controlled trials addressing these questions. However, we chose to avoid observational studies to improve the strength of our causal conclusions.

### Information sources

We will search general databases (PubMed, Embase and Scopus) and databases specific to allied health (Cumulative Index to Nursing and Allied Health Literature (CINAHL), Physiotherapy Evidence Database (PEDro) and SPORTDiscus) as well as Cochrane Central Register of Controlled Trials, Cochrane Database of Systematic Reviews and Clinicaltrials.gov registry.

Databases will be searched from inception up to the current date for peer-reviewed articles. Reference lists of the included studies and relevant systematic reviews will be manually searched to identify any relevant articles. In addition, forward citation searching of included studies will be undertaken in Google Scholar and Scopus.

When full texts are unavailable or in the case of missing relevant data, the authors will be approached via email to obtain this information. No language restrictions shall be applied to our search. The services of a professional translator will be sought for articles that are not available in the English language.

### Search strategy and study selection

The search strategy was designed by review authors in consultation with a professional librarian and pretested prior to the formal search. Key terms pertaining to “exercise”, “HIV”, “adults” and “interventional study” were included; see details in Table 2. Outcomes were not included in the search strategy to improve the search’s reach. Records retrieved will be exported to and managed in the EndNote referencing software. After elimination of duplicates, screening will be performed by one reviewer and a fraction of the records repeated independently by a second. Studies with irrelevant titles will be excluded. Abstracts of the remaining studies will then be examined using a pre-established inclusion checklist. Where additional information is required to determine eligibility, the reviewers will contact the authors concerned via email. Where there are several publications of the same study (such as a protocol paper cited in the principal article), the publications will be collated and considered together. When abstracts are deemed relevant or where eligibility is unclear, full texts will be obtained. Full texts will be assessed independently by two review authors in the same manner as the initial screen. Any disagreements will be resolved through consensus or by the decision of a third independent reviewer.

**Table 2:** PubMed search strategy

***Field limits***	#1 ***title and abstract***	#4 ***title and abstract***	#9 ***title and abstract***	#12 ***title and abstract***
**Keyword query**	"human immunodeficiency" OR "human immune deficiency" OR HIV OR "acquired immune deficiency" OR "acquired immunodeficiency" OR "aquired immunodeficiency"	exercis* OR sport* OR workout OR physiotherapy OR kinesiotherapy OR walking OR jogging OR running OR swimming OR bicycling OR cycling OR "weight-lifting" OR dancing OR “resistance bands” OR telerehabilitation OR “physical activity” OR "physical therapy"	individuals OR women OR men OR patients OR participants OR people OR adults OR females OR males OR subjects OR persons OR elderly OR volunteers	Intervention OR program OR trial
		**#5** ***title and abstract***		
		(physical OR aerobic OR resistance) AND (fitness OR training OR rehabilitation)		
		**#6** ***title and abstract***		
		(weight OR endurance OR strength OR circuit) AND training		
	**#2**	**#7**	**#10**	**#13**
**Index term query**	"HIV" OR "HIV Long-Term Survivors" OR "HIV Infections"	"Exercise" OR "Exercise Therapy" OR "Sports" OR "Exercise Movement Techniques"	“adults”	"Clinical Trials as Topic" OR "Interrupted Time Series Analysis" OR "Controlled Before-After Studies" OR "Historically Controlled Study" OR "Clinical Trial" OR "Systematic Review"
**Combined**	**#3 = #1 OR #2**	**#8 = #4 OR #5 OR #6 OR #7**	**#11 = #9 OR #10**	**#14 = #12 OR #13**
**Final string**	**#3 AND #8 AND #11 AND #14**			

### Data extraction and management

A pre-piloted data extraction form developed in Microsoft Excel by the authors will be used to extract information (see form attached (Additional file 2)). The data extraction form will be pretested and calibrated by a group of two reviewers, to promote agreement and reduce the risk of subjective misclassification. Study characteristics and outcome measures will then be extracted by one reviewer and randomly cross-checked by a second. Disputes between the two extractors will be resolved by consensus or the decision of a third reviewer. We will attempt to obtain missing information from supplementary files if available (such as public data repositories) or contact the authors via email.

Study characteristics extracted will include study date, study aim/objectives, setting of the study, description of participants and study design including recruitment procedure. Details of the home/community exercise intervention and the outcomes of interest including adherence will equally be extracted. In addition, the details of the descriptions of comparators for all studies will be extracted (Additional file 2). These general characteristics will be summarized and presented in a table in the results.

### Quality assessment

Two reviewers will independently assess the quality of included studies. Disputes between the two assessors will be resolved by consensus or the decision of a third assessor. Comprehensive reporting and risk of bias in individual studies will be assessed using version 2 of the Cochrane risk-of-bias tool for randomized trials (RoB 2) [35] and the Risk of Bias In Non-Randomized Studies-of Interventions (ROBINS-I) tool for non-randomized concurrently controlled and single-arm interventional studies [36]. The RoB 2 tool assesses bias across 5 domains which are randomization, deviations from the intended intervention, missing outcome data, measurement of the outcome and selection of the reported results. Through well-described algorithms [35], the reviewer is guided to make a judgement for each domain, as well as an overall risk-of-bias judgement, assigning low risk of bias, some concerns or high risk of bias. The ROBINS-I tool for non-randomized studies of interventional studies is a multiple-domain tool which covers confounding, selection of participants, information bias, deviations from the intended intervention, missing data, outcome measurement and selection of the reported results. As with the RoB 2 tool, through established algorithms [36], the reviewer is guided to make a judgement for each domain, assigning low risk of bias, moderate risk, serious risk, critical risk or no information [36], as well as an overall risk-of-bias judgement, assigning low risk of bias, moderate risk, serious risk or critical risk.

Assessments will be performed for each of the primary outcomes evaluated in the study separately. We will perform an overall assessment of risk per outcome and per study and summarize in a risk of bias table.

### Data analysis, assessment of heterogeneity and publication bias

Exercise volume will be translated to metabolic equivalent (MET) hours per week based on standard definitions in the compendium of physical activities [37], to ease comparison across studies. In studies with multiple intervention groups, we shall report outcomes for all groups relative to the outcome in the minimally supervised home exercise group. Where a study reports outcome at multiple time points, we will consider the timepoints that immediately precedes and the timepoint that immediately follows the intervention in our analysis.

Continuous outcome measures will be reported as mean difference (final minus baseline) or standardized mean differences where there are different scales for the same outcome. In the event where authors report medians and interquartile ranges, we will use this to compute corresponding means and standard deviations as described in previous literature [38]. Where possible, missing outcomes will be computed from other reported statistics such as percentage body fat from skinfold thickness using validated formulae [39].

Where there are at least two studies with common or similar primary outcome measures (reported or computed) and study design, we will undertake a quantitative synthesis. We will use Comprehensive Meta-Analysis Software (CMA) software, to pool mean differences for each primary outcome variable using inverse variance weighting (or if different scales for the same outcome, standardized mean effect sizes). We will perform separate pooling of effect sizes for randomized studies, non-randomized multiple group studies and single-arm studies. We anticipate some heterogeneity in participant characteristics, study quality, sample sizes, and type and volume of exercise; hence, we will assume and conduct a random effects meta-analysis. Where possible, the intervention effects adjusted for the highest number of confounding factors will be used in the synthesis of non-randomized studies. Non-randomized studies assessed to be at critical risk of bias will be excluded from quantitative synthesis. The Cochran’s *Q* test will be used to assess for heterogeneity and the *I*^2^ statistic to quantify it. *I*^2^ of 0–30% will be considered minimal, 30–55% moderate, 55–75% substantial heterogeneity and 75–100% considerable heterogeneity [40].

Sensitivity analysis will be carried out to restrict the analysis to studies with low risk of bias, studies without co-interventions and studies that performed comparisons with “no exercise” and to assess the impact of these on the effect sizes.

We will perform subgroup analysis (or a narrative synthesis if scarce studies) to compare the effect size for primary outcomes across studies with “minimal supervision” versus “no supervision” and “technology-assisted delivery” versus “no technology” as well as by type and intensity of exercise. We hypothesize that interventions with some professional contact and those with technological assistance will perform better than interventions where these features are absent.

Publication bias will be explored using funnel plots [41, 42] and tested using Egger’s [43] regression test. Where either of these suggests significant bias, we will attempt to correct this using the trim and fill method of Duval and Tweedie [44] and report both the original and updated random effects estimates after accounting for publication bias.

Our findings will be summarized by considering the five Grading of Recommendations Assessment, Development and Evaluation (GRADE) criteria (study limitations, inconsistency of effect, imprecision, indirectness of evidence and publication bias) as outlined previously [36] to determine the strength and quality of evidence for each primary outcome.

Secondary outcomes will be synthesized narratively and discussed in relation to changes in the primary outcomes of interest.

## Discussion and conclusion

The review will shed light on the effectiveness of self-managed home/community exercise in improving adiposity indices and hence preventing cardiometabolic complications in PLWH. We will equally demonstrate the benefits (if any) of technological assistance in such exercise programmes.

In this proposal, we describe how we will calculate exercise volumes for each study and hence address the variability in exercise prescription. We equally outline the steps we will take to synthesize our outcomes to arrive at meaningful comparisons. Any changes made in the methods we describe here will be documented and reported in a follow-up manuscript with our results.

Considering that a review of this kind is lacking for PLWH, we believe that our methods and eventual findings are filling an important knowledge gap. In addition to publishing our findings in a scientific journal, this work will be included in the thesis of the principal investigator and made available to the public by her institution.

## Supplementary Information

**Additional file 1.** Preferred Reporting Items for Systematic reviews and Meta-analysis Protocols (PRISMA-P) Checklist. Microsoft word document with populated checklist adapted from PRISMA-P and the PRISMA 2020 for Abstracts Checklist.

**Additional file 2.** Data Extraction Sheet. Microsoft Excel document with detailed screening and extraction items.

## Data Availability

Not applicable

## Abbreviations

HIV: Human immunodeficiency virus
ART: Antiretroviral therapy
PLWH: People living with HIV
PROSPERO: Prospective Register of Systematic Reviews
BMI: Body mass index
FMI: Fat mass index
BAI: Body adiposity index
WHR: Waist-to-hip ratio
VAT: Visceral adipose tissue
CINAHL: Cumulative Index to Nursing and Allied Health Literature
PEDro: Physiotherapy Evidence Database
EPOC: Effective Practice and Organisation of Care
MET: Metabolic equivalent
CMA: Comprehensive Meta-Analysis Software
PRISMA-P: Preferred Reporting Items for Systematic reviews and Meta-analysis Protocols
GRADE: Grading of Recommendations Assessment, Development and Evaluation
